# Supplementary material for: The interactome of intact mitochondria by cross-linking mass spectrometry provides evidence for coexisting respiratory supercomplexes
Source: Mol Cell Proteomics. 2017 Dec 8;17(2):216–32. doi: 10.1074/mcp.RA117.000470 (PMC5795388; doi:10.1074/mcp.RA117.000470)
Supplement: Supplemental Data [file supp_RA117.000470_133922_0_supp_23978_fzffwf.zip › spectra_annotation/mito_DR_spectra_annotation/212-1-7-1-16-1.pdf]

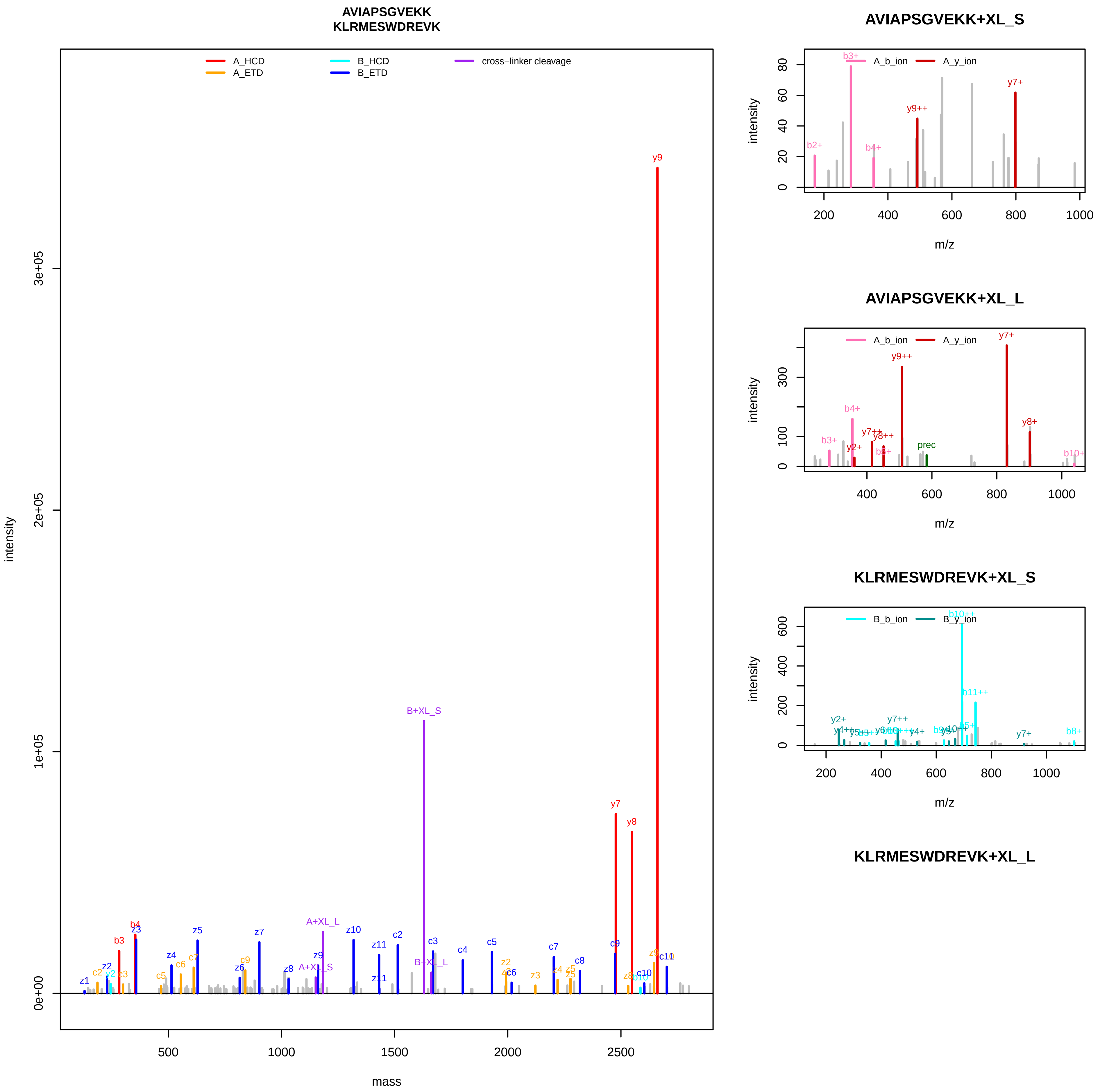
AVIAPSGVEKK
KLRMESWDREVK
A_HCD
A_ETD
B_HCD
B_ETD
cross−linker cleavage
intensity
mass
0e+00
1e+05
2e+05
3e+05
500
1000
1500
2000
2500
y9
y7
y8
B+XL_S
A+XL_L
A+XL_S
B+XL_L
b3
b4
z1
z2
z3
z4
z5
z6
z7
z8
z9
z10
z11
c2
c3
c4
c5
c6
c7
c8
c9
c10
c11
AVIAPSGVEKK+XL_S
A_b_ion
A_y_ion
b2+
b3+
b4+
y9++
y7+
intensity
m/z
AVIAPSGVEKK+XL_L
A_b_ion
A_y_ion
b3+
b4+
y2+
y7++
y8++
y9++
prec
y7+
y8+
b10+
intensity
m/z
KLRMESWDREVK+XL_S
B_b_ion
B_y_ion
y2+
y7++
b10++
b11++
y7+
b8+
intensity
m/z
KLRMESWDREVK+XL_L
